# The Traditional Chinese Medicine Fufang Shatai Heji (STHJ) Enhances Immune Function in Cyclophosphamide-Treated Mice

**DOI:** 10.1155/2020/3849847

**Published:** 2020-01-23

**Authors:** Kai-Jian Fan, Yun-Wu Li, Jing Wu, Jun Li, Jun Zhang, Qi-Shan Wang, Bing-Xin Xu, Qing Cai, Ting-Yu Wang

**Affiliations:** ^1^Department of Pharmacy, Shanghai Ninth People's Hospital, School of Medicine, Shanghai Jiao Tong University, Shanghai 200011, China; ^2^Department of Pharmacy, Mental Health Center, Chongming District, Shanghai 202150, China; ^3^Department of Orthopedic Surgery, Rush University Medical Center, Chicago, IL 60612, USA; ^4^Department of Internal Medicine, Division of Nephrology, University of California at Davis, Davis, CA 95616, USA; ^5^Department of Immunology and Rheumatology, Changhai Hospital, The Second Military Medical University, Shanghai 200433, China

## Abstract

Fufang Shatai Heji (STHJ) is a mixture of traditional Chinese medicines, such as *Radix Adenophorae*, *Radix Pseudostellariae*, and *Radix Astragali*. STHJ is commonly used to treat diseases caused by low immune function, for example, Sjögren's syndrome (SS). The primary objective of this study was to assess the immunopotentiating effect of STHJ using an immunosuppressive mouse model receiving cyclophosphamide (CTX). Following CTX treatment, STHJ was administered by oral gavage for 30 consecutive days. The percentage of specific lymphocyte subpopulations in the spleen was measured by flow cytometry. Levels of inflammatory factors in serum were detected by enzyme-linked immunosorbent assays (ELISAs). The administration of STHJ significantly elevated thymus and spleen indices, increased B cell and natural killer (NK) cell activities, and decreased CD8^+^ T, CD8^+^CD122^+^ T, NKT, and *γδ*T cell activities in the CTX-treated mice. In addition, STHJ upregulated the expression of interleukin- (IL-) 2, IL-6, and tumor necrosis factor-*α* (TNF-*α*) and downregulated IL-10 expression in CTX-treated mice. In conclusion, STHJ effectively remitted CTX-induced immunosuppression by modulating the balance of lymphocyte subsets and cytokines. Our results suggest STHJ treatment could be used as an effective therapeutic approach to improve immune function in patients with low immunity.

## 1. Introduction

Job-related pressures and environmental pollution have been identified as the leading causes of immune suppression in the working population. Reduced immune strength could increase the risk of infection leading to autoimmune diseases. A previous study found that significant changes in the population of immune cells, such as neutrophils, lymphocytes, and eosinophils, would result in an impaired immune system [1]. To meet the clinical need, it is necessary to develop novel treatment strategies to improve immune function.

Cyclophosphamide (CTX) is a chemotherapeutic drug primarily used to treat tumors. It has been shown to have strong immunosuppressive effects by decreasing the absolute number of T cells, circulating B cells and synthesis of IgG. A number of studies have shown that animals treated with CTX could be used as a suitable model of long-term immunosuppression to evaluate the immune enhancement effects of drugs [2]. Previous studies found significant changes in both cellular and humoral immunity in CTX-induced immunosuppressed mice with reduced CD4^+^/CD8^+^ T cell ratios and B cell numbers [3]. Several immune cell subsets, such as immunosuppressive T regulatory cells (Tregs) and natural killer T (NKT) cells, have recently been identified and have been shown to play important roles in maintaining the balance of immunity. Whether these immunosuppressive cell subsets are affected in CTX-induced immunosuppressed mice and whether drugs could improve immunity through regulating these cells need to be further investigated.

Traditional Chinese medicine (TCM) with low toxicity could be administered for long periods of time to treat chronic diseases with low immune activities. Fufang Shatai Heji (also known as SS syrup, STHJ) is a concentrated liquid obtained by secondary decoction of various Chinese herbal medicines. These Chinese herbal medicines mainly include *Pseudostellaria heterophylla* (Miq.) Pax, root (*Radix Pseudostellariae*); *Adenophora tetraphylla* (Thunb.) Fisch., root (*Radix Adenophorae*); *Astragalus membranaceus* (Fisch.) Bge., root (*Radix Astragali*); *Ophiopogon japonicus* (Thunb.) Ker Gawl., root (*Radix Ophiopogonis*); *Dendrobium nobile* Lindl., stem (*Dendrobium nobile*); *Glycyrrhiza uralensis* Fisch., and rhizome (*Glycyrrhiza uralensis*). *Radix Pseudostellariae* contains polysaccharides and amino acids and has antitumor and immune-enhancing effects [4]. *Radix Adenophorae* largely contains polysaccharides and coumarin, which regulates immune function and eliminates free radicals [5]. The main components of *Radix Astragali* are Astragalus polysaccharides and Baicalin. Among these components, Astragaloside IV has been shown to have immunomodulatory effects by regulation of the activity of protein tyrosine phosphatase CD45 [6]. STHJ has been effectively used in the clinic to improve the immunity of patients for more than 30 years. A number of clinical studies have also confirmed the immunomodulatory effects of STHJ [7, 8]. Moreover, many published papers have demonstrated the immunomodulatory effects of ingredients of STHJ. Yang found that the extract of supercritical carbon dioxide from *Radix Adenophorae* significantly increased the absolute number of CD3^+^T, CD4^+^T, and CD8^+^ T cells in the peripheral blood of immunosuppressed mice, thereby producing a significant recovery effect on the peripheral immune system [9]. Wang et al. found that the extract of Radix Pseudostellariae has an obvious antagonistic effect on the low conversion of T and B lymphocytes and a decrease of the phagocytic function of leukocytes induced by CTX [10]. Shi et al. found that Astragalus polysaccharide can significantly increase the phagocytosis of peritoneal macrophages and the conversion rate of peripheral blood lymphocytes in immunosuppressed mice induced by CTX [11]. In a preliminary animal experiment, we found STHJ could regulate the immunity of mice (unpublished data).

In the present study, we investigated the effects of STHJ on immune function in CTX-treated immunosuppressed mice. Bailing capsule was used as the positive drug control because it is often used to improve immunity in the clinic. The ratio of lymphocyte subpopulations and the balance between inflammatory and anti-inflammatory factors were detected to investigate whether STHJ could serve as a potential therapeutic agent to improve immunity.

## 2. Materials and Methods

### 2.1. Animals

Balb/c mice (male, 18–22 g, 6–8 weeks old) were purchased from the Laboratory Animal Center of Shanghai Ninth People's Hospital. The mice were kept in SPF facilities with pathogen-free conditions (temperature, 22°C ± 2°C; humidity, 55% ± 5%) with a 12 h light-dark cycle. The experimental protocol was approved by the Ethics Committee of the Ninth People's Hospital affiliated with the Shanghai Jiao Tong University School of Medicine.

### 2.2. Drugs and Reagents

STHJ was obtained from the Shanghai Ninth People's Hospital (Shanghai, China). Briefly, *Radix Pseudostellariae* 300 g, *Radix Adenophorae* 120 g, *Radix Astragali* 120 g, *Radix Ophiopogonis* 90 g, *Dendrobium nobile* 90 g, and *Glycyrrhiza Uralensis* 30 g were combined and extracted by boiling with an eight times volume of water for 1.5 hours and then a six times volume of water for 1 hour. Each extract was collected after boiling and filtered. The two filtrates were combined and let stand for 12 hours. The filtrate was then concentrated to 1000 ml as STHJ. Injectable cyclophosphamide (CTX) was purchased from Baxter Oncology GmbH (Halle, Germany). Bailing was used as the positive control and purchased from Huadong Medicine Co. Ltd (Hangzhou, China). Mouse anti-CD3e-APC, mouse anti-CD4-FITC, mouse anti-CD8a-PE, mouse anti-Foxp3-PE, mouse anti-*γδ*T-FITC, mouse anti-CD45R-PE, mouse anti-CD49b-FITC, mouse anti-CD122-APC, Foxp3/transcription factor staining buffer set, and cell stimulation cocktail were purchased from eBioscience (San Diego, CA, USA). Mouse ELISA kits (TNF-*α*, IL-2, IL-6, and IL-10) were purchased from Multi Sciences (Hangzhou, China).

### 2.3. Experimental Protocols

Sixty mice were randomly assigned into five groups of 12 mice. One group was used as the normal control group; the others were intraperitoneally injected with CTX (80 mg/kg/d) for three consecutive days. From day 4, three groups of CTX-treated mice were infused by oral gavage once a day for 30 days as follows: low STHJ group (10 ml/kg, low dosage of STHJ), high STHJ group (20 ml/kg, high dosage of STHJ), and the Bailing group (1.2 g/kg, Bailing). The mice in the CTX-induced model group were treated with PBS by the same way.

### 2.4. Analysis of Astragaloside IV in STHJ by Thin-Layer Chromatography (TLC)

STHJ (50 ml) was extracted three times with 50 ml of water-saturated *n*-butanol; the *n*-butanol extracts were then combined. After which, the resulting extract was washed three times with 50 ml ammonia water and three times with 50 ml distilled water. The *n*-butanol extract was evaporated to dryness, and the residue was dissolved in methanol (1 ml) to obtain the test solution. The Astragaloside IV reference substance (1 mg) was dissolved in methanol (1 ml) to obtain the standard solution. A solution of chloroform-methanol-water (13 : 7 : 2) was mixed and placed at 10°C; the lower layer solution was used as the developing solvent. According to the thin-layer chromatography method, 5 *μ*l of each of the standard solution and the test solution was placed on the same silica gel G thin-layer plate and placed in a chromatographic tank for development. At the end of expansion, the plate was removed from the tank and dried, then sprayed with 10% sulfuric acid ethanol solution, and heated at 105°C until the spots were visible.

### 2.5. Effect of Drugs on Spleen and Thymus Indices

At the end of the experiment at 30 days, all mice were anesthetized with an intraperitoneal injection of sodium pentobarbital (45 mg/kg). The anesthetized mice were weighed and then killed by cervical dislocation. The spleen and thymus were collected and weighed to calculate the spleen and thymus indices, which were calculated as follows: spleen index (%) = (spleen weight/body weight) × 100% and thymus index (%) = (thymus weight/body weight) × 100%.

### 2.6. Isolation of Splenocytes

The spleen was obtained from the mice sacrificed under aseptic conditions, washed with sterile PBS, and crushed to isolate the splenocytes. The splenocytes were passed through a 100-mesh filter to obtain a uniform cell suspension. The obtained cell suspension was centrifuged at 2000 rpm for 5 minutes. The reclaimed splenocytes were resuspended in red blood cell lysis buffer for 5 minutes to remove red blood cells. After centrifugation, the splenocytes were resuspended in PBS and then filtered. The splenocyte suspension was obtained by resuspending again after centrifugation.

### 2.7. Determination of the Proportion of Lymphocyte Subsets in Splenocytes

For the determination of surface antigens, splenocytes were treated with three staining combinations. 100 *μ*l of the splenocyte suspension prepared above was incubated for 30 minutes at 4°C in the dark with the following: 1.25 *μ*l of APC-CD3^+^, 0.5 *μ*l of FITC-CD4^+^, and 1.25 *μ*l of PE-CD8^+^; or 1.25 *μ*l of APC-CD3^+^, 1 *μ*l of FITC-CD49b^+^, and 2.5 *μ*l of PE-CD45R^+^; or 1.25 *μ*l of PE-CD8^+^, 1.25 *μ*l of APC-CD122^+^, and 1 *μ*l FITC-*γδ*T^+^. For the determination of Treg, the surface antigen of CD4^+^ was labeled first and intracellular staining with PE-Foxp3^+^ was performed after fixation and permeabilization. Following the incubations, the cells were washed twice with PBS and resuspended in PBS. The percentages of lymphocytes were analyzed by flow cytometry (Beckman Coulter CytoFlex S).

### 2.8. Measurement of Serum Cytokines by ELISA

At the end of the experiment, as stated above, all mice were anesthetized with an intraperitoneal injection of sodium pentobarbital (45 mg/kg). Blood was collected from the angular vein of the eyes of the anesthetized mice. Serum was obtained after centrifugation of blood at 3600 rpm for 10 minutes at 4°C. Cytokine (TNF-*α*, IL-2, IL-6, and IL-10) levels in the serum were measured using commercial ELISA kits, according to the manufacturer's instructions.

### 2.9. Statistical Analysis

The results are expressed as mean ± standard deviation (SD). A one-way analysis of variance (ANOVA) followed by Bonferroni test was used for multiple comparisons between various groups. Differences between groups with *P* < 0.05 are considered statistically significant.

## 3. Results

### 3.1. TLC Analysis of STHJ Extract

**Figure 1:**
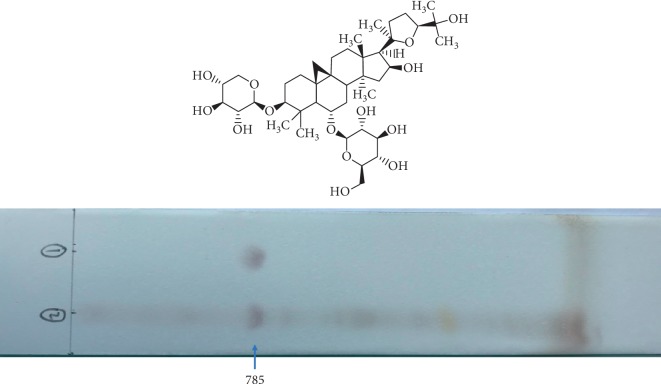
Thin-layer chromatogram (TLC) of the extraction solutions of the standard (Astragaloside IV) [1] and the mixture of traditional Chinese medicines, Fufang Shatai Heji (STHJ) [2].

Astragaloside IV is the dominant component of SHTJ. In the chromatogram, the STHJ sample and the standard appeared in corresponding positions and showed spots of the same color (Figure 1).

### 3.2. Effect of STHJ on the Spleen and Thymus Indices in CTX-Treated Mice

**Figure 2:**
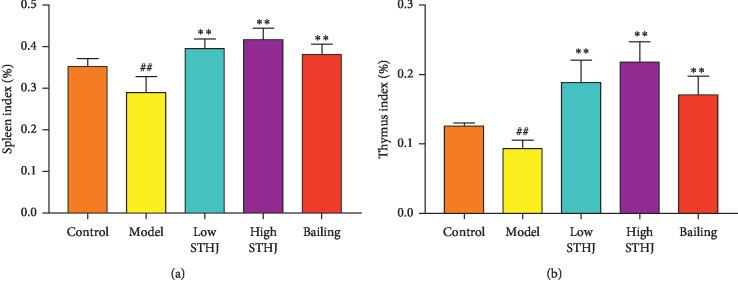
Effect of the mixture of traditional Chinese medicines, Fufang Shatai Heji (STHJ), on the indices of immune organs in cyclophosphamide- (CTX-) treated mice. Sixty mice were randomly assigned into five groups of 12 mice. One group was used as the normal control group, and the others were intraperitoneally injected with CTX (80 mg/kg/d) for three consecutive days. Animals in the model group were treated with PBS. The remaining three groups were infused by oral gavage once a day for 30 days with low STHJ (10 ml/kg), high STHJ (20 ml/kg), and the Bailing group (1.2 g/kg). (a) Spleen index and (b) thymus index. All data are expressed as mean ± standard deviation (SD) (*n* = 12). ^##^*P* < 0.01 vs. control group; ^*∗∗*^*P* < 0.01 vs. model group.

To investigate the immune regulatory effects of STHJ, low and high doses of STHJ were orally administered to CTX-induced immunosuppressed mice for 30 consecutive days. Compared to the control group, the spleen and thymus indices decreased significantly in the CTX-induced model group (*P* < 0.01, Figure 2). In contrast, the spleen and thymus indices were significantly higher in both STHJ and Bailing groups than that in the model group. These data demonstrate that STHJ had a significant protective effect on the reduction of spleen and thymus indices by CTX.

### 3.3. Effect of STHJ on NK Cells in CTX-Treated Mice

**Figure 3:**
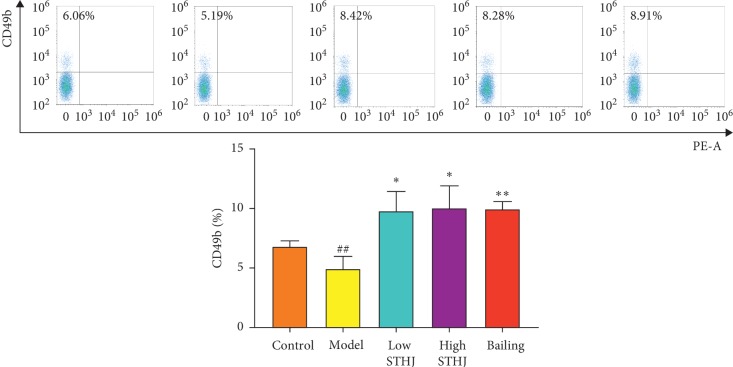
Influence of the mixture of traditional Chinese medicines, Fufang Shatai Heji (STHJ), on natural killer (NK) cells in cyclophosphamide- (CTX-) treated mice. NK cells were labeled with CD49b. STHJ significantly enhanced the activity of NK cells without dose dependence. All data are expressed as mean ± standard deviation (SD) (*n* = 12). ^##^*P* < 0.01 vs. control group; ^*∗∗*^*P* < 0.01 vs. model group.

NK cells are important components of the innate immune system and are involved in a variety of diseases, including autoimmune diseases. In this study, CTX injection significantly inhibited the percentage of NK cells in splenocytes of mice (*P* < 0.01, Figure 3). In comparison with those in CTX-treated mice, increased NK cell percentages were observed in splenocytes isolated from mice treated with different concentrations of STHJ. A more pronounced effect was observed in the high-dose STHJ group (*P* < 0.01).

### 3.4. Influence of STHJ on T Cells in CTX-Treated Mice

**Figure 4:**
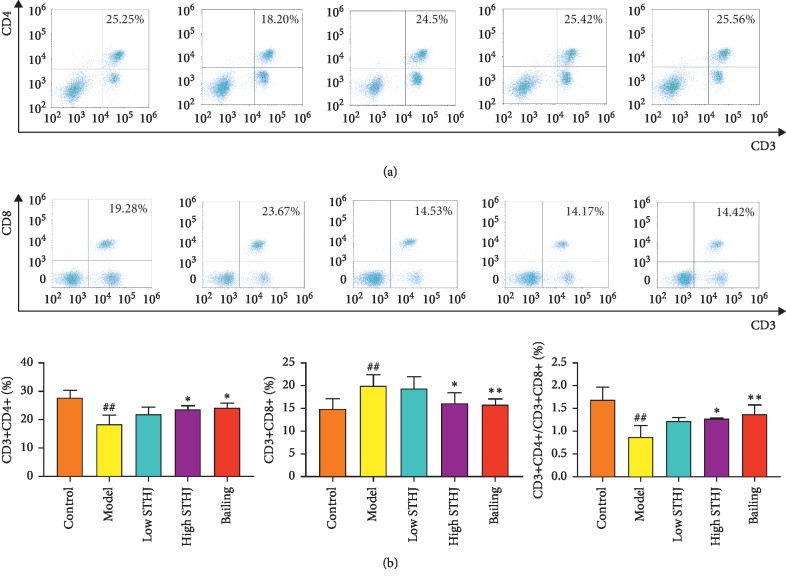
Influence of the mixture of traditional Chinese medicines, Fufang Shatai Heji (STHJ), on T cells in cyclophosphamide- (CTX-) treated mice. (a, b) The high-dose of STHJ significantly altered the activity of CD4^+^ T and CD8^+^ T cells. All data are expressed as mean ± standard deviation (SD) (*n* = 12). ^##^*P* < 0.01 vs. control group; ^*∗∗*^*P* < 0.01 vs. model group.

Treatment with CTX significantly inhibited the percentage of CD4^+^ T cells and increased the percentage of CD8^+^ T cells (*P* < 0.01, Figures 4(a) and 4(b)). Compared to CTX treatment alone (model group), the administration of low-dose STHJ showed no significant effects on the percentage of CD4^+^ or CD8^+^ T cells, but the high-dose STHJ and Bailing significantly increased the percentage of CD4^+^T cells and decreased the percentage of CD8^+^ T cells. In addition, the ratio of CD4^+^/CD8^+^ T cells was significantly increased in the high-dose STHJ group and the Bailing group as compared to the CTX treatment only model group.

### 3.5. Effect of STHJ on T Lymphoid Subpopulations in CTX-Treated Mice

**Figure 5:**
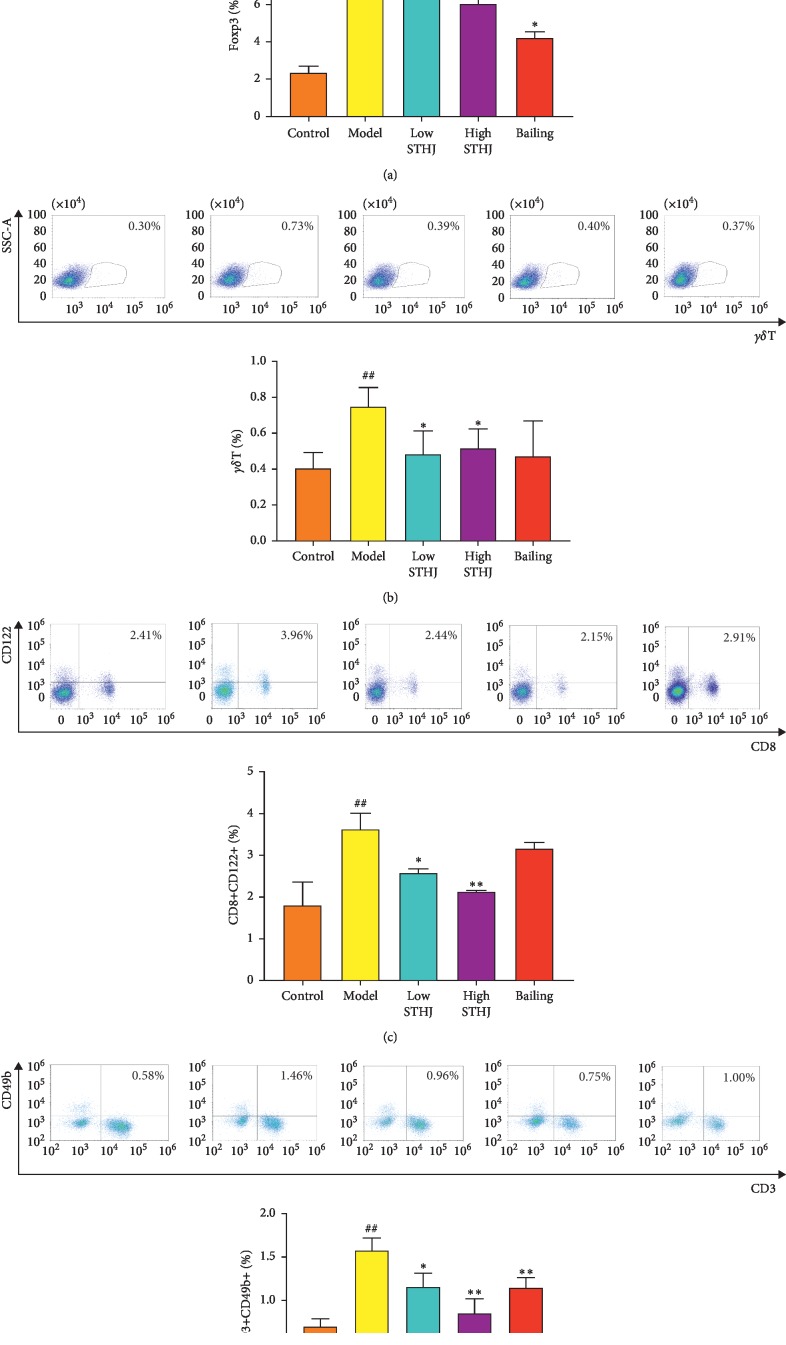
Effect of the mixture of traditional Chinese medicines, Fufang Shatai Heji (STHJ), on inhibitory T cells in cyclophosphamide- (CTX-) treated mice. (a) Treg cells were labeled with CD4^+^Foxp3^+^; STHJ had no significant effect on Treg cells. (b) At all doses, STHJ produced a remarkable downregulation of *γδ*T cell activities. (c) STHJ significantly downregulated the activities of CD8^+^CD122^+^ T and its effect on CD8^+^CD122^+^ T was dose-dependent. (d) Natural killer T (NKT) cells were labeled with CD3^+^CD49b^+^. STHJ significantly inhibited the activity of NKT cells, and this effect was especially pronounced in the high-dose STHJ group. All data are expressed as mean ± standard deviation (SD) (*n* = 12). ^##^*P* < 0.01 vs. control group; ^*∗∗*^*P* < 0.01 vs. model group.

During immune homeostasis, T lymphoid subpopulations play a key role in modulating immune status. In recent years, several T lymphoid subpopulations have been identified and studied. In this study, we detected the percentages of Treg, *γδ*T, CD8^+^CD122^+^T, and NKT cells in the spleen. Compared to normal control mice, CTX treatment elicited a marked increase in the proportion of several inhibitory T cells (*P* < 0.01, Figures 5(a)–5(d)). Compared to CTX-treated mice, neither low- nor high-dose STHJ had any significant influence on Treg cells (Figure 5(a)), but Bailing treatment significantly suppressed the proportion of Treg cells (*P* < 0.05). STHJ in both low- and high-dose groups showed a remarkable downregulation in the percentage of *γδ*T cells (*P* < 0.05, Figure 5(b)). In contrast, Bailing showed no significant effect on *γδ*T cells. Treatment with STHJ significantly downregulated the percentage of CD8^+^CD122^+^ T cells; this effect was more pronounced in the high-dose STHJ group (*P* < 0.01, Figure 5(c)). However, Bailing had no significant effect on CD8^+^CD122^+^ T cells. Treatment with STHJ or Bailing significantly downregulated the percentage of NKT cells; this effect was especially pronounced in the high-dose STHJ group (*P* < 0.01, Figure 5(d)).

### 3.6. Effect of STHJ on B Cells in CTX-Treated Mice

**Figure 6:**
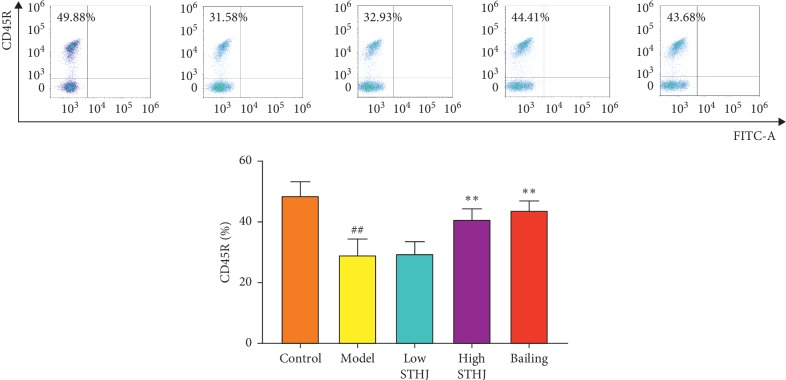
Effect of the mixture of traditional Chinese medicines, Fufang Shatai Heji (STHJ), on B cells in cyclophosphamide- (CTX-) treated mice. B cells were labeled with CD45R. High-dose STHJ significantly enhanced the activity of B cells. All data are expressed as mean ± standard deviation (SD) (*n* = 12). ^##^*P* < 0.01 vs. control group; ^*∗∗*^*P* < 0.01 vs. model group.

B cells and their antibodies are important factors in humoral immunity and the immune response. CTX injections significantly inhibited B cells in the splenocytes of mice (*P* < 0.01, Figure 6). Compared to CTX-treated mice, the percentage of B cells was not altered in the low-dose STHJ group, but was significantly increased in the high-dose STHJ group or Bailing group (*P* < 0.01).

### 3.7. Effect of STHJ on Serum Cytokine Levels in CTX-Treated Mice

**Figure 7:**
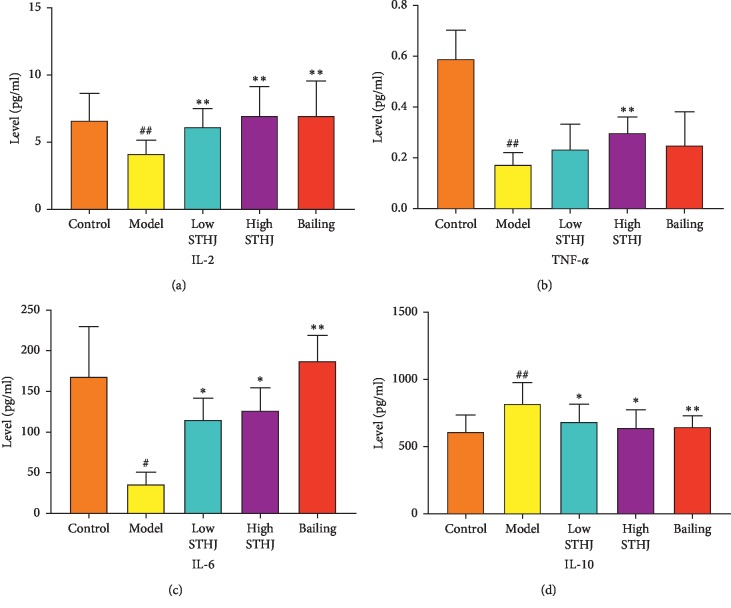
Effect of the mixture of traditional Chinese medicines, Fufang Shatai Heji (STHJ), on the serum levels of interleukin-2 (IL-2), IL-6, IL-10, and tumor necrosis factor-*α* (TNF-*α*) in cyclophosphamide- (CTX-) treated mice. All data are expressed as mean ± standard deviation (SD) (*n* = 12). ^##^*P* < 0.01 vs. control group; ^*∗∗*^*P* < 0.01 vs. model group.

Compared to those in the normal control group, serum levels of TNF-*α*, IL-2, and IL-6 decreased significantly while the levels of IL-10 increased significantly in CTX-treated mice (Figure 7). With STHJ or Bailing treatments, IL-2 and IL-6 levels were markedly upregulated (*P* < 0.05 or *P* < 0.01). Treatment with STHJ or Bailing significantly downregulated IL-10 levels in serum (*P* < 0.05 or *P* < 0.01). High-dose STHJ induced a significant increase in serum levels of TNF-*α*, but Bailing failed to produce any significant effect on TNF-*α* levels.

## 4. Discussion

CTX and glucocorticoids are two of the most commonly used immunosuppressive drugs in animal studies. Although CTX is a drug primarily used to treat tumors, it has recently been shown to have strong immunosuppressive effects [12]. Glucocorticoids exert inhibitory effects on various immune processes and are, thus, used as antiallergic or anti-inflammatory drugs [13]. Although both CTX and glucocorticoids have immunosuppressive effects, glucocorticoids show no significant effects on B cells. On the other hand, CTX is an efficient drug to induce long-term immunosuppression. Therefore, the CTX-induced immunosuppression mouse model is a suitable model for evaluating the immunomodulatory effects of drugs [2]. Bailing capsule is obtained by fermentation of artificially cultivated fungal strains. It is an asexual type of Cordyceps sinensis, which has similar chemical composition to the natural form. The ingredients of Bailing capsule mainly include adenosine, a variety of amino acids, a variety of vitamins, and trace elements; it can be used as a substitute for natural Cordyceps. Recent studies have shown that Bailing capsule has the effect of increasing immune function [14]. Due to its good immune enhancement, Bailing capsule was used as a positive control drug in this study.

The spleen and thymus are important immune organs where lymphocytes differentiate, mature, and initiate immune responses. The functional state of immune organs directly affects the immune function in the body. When the body is immunocompromised, the spleen and thymus shrink. We found that STHJ significantly increased spleen and thymus indices of immunosuppressed mice, indicating that STHJ has certain immune enhancement functions. To investigate the mechanisms associated with this increase, we conducted a more in-depth in vivo study on the effects of STHJ on lymphocytes and inflammatory factors.

Immunity plays a very important role in the body against disease, mainly comprised of innate and adaptive immunities. The innate immune response is the initial line of defense for the body and is nonspecific. Innate immune cells primarily include phagocytic cells, dendritic cells, and NK cells. NK cells act principally through their secreted cytokines and their natural toxicity to target cells [15–17]. For example, TNF-*α* and IL-10 secreted by NK cells could regulate immune function by affecting downstream immune responses [17, 18]. TNF-*α* is a potent proinflammatory cytokine that is involved in many immune functions. For these reasons, anti-TNF-*α* antibodies are used to treat some autoimmune diseases, such as rheumatoid arthritis (RA) [19]. In addition, several studies have found that NK cells inhibit the function of CD4^+^ and CD8^+^ T cells [20–23] while Treg cells in turn inhibit the activity of NK cells [24]. Thus, NK cells are thoroughly involved in immune regulation. Using immunosuppression models, Jang et al. reported a significant reduction in the number of NK cells in the spleen of CTX-treated mice [25]. In this study, the proportion of NK cells in the CTX-treated mice was also reduced, and this effect was reversed by STHJ treatment at both low and high doses. Therefore, we conclude that NK cells are sensitive to STHJ and are involved in the mechanism of STHJ-enhanced immune functions.

The adaptive immune system is mainly composed of T and B lymphocytes that mediate cellular and humoral immunity, respectively. The main types of T cells are CD4^+^ and CD8^+^ T lymphocytes, which are the key regulators in adaptive immune responses [26]. An imbalance in the ratio of CD4^+^ T lymphocytes to CD8^+^ T lymphocytes may lead to the growth of autoimmune diseases. Here, we first examined the effect of STHJ on total T lymphocytes. Using the CTX-induced immunosuppression mouse model, the results in our study showed that the CD4^+^/CD8^+^ T cell ratio was significantly reduced in the CTX-treated mice, which is consistent with that reported by Yu et al. [3]. Interestingly, we found that high dose of STHJ significantly inhibited the proliferation of CD8^+^ T cells and increased the number of CD4^+^ T cells, resulting in an increased CD4^+^/CD8^+^ ratio. It has been reported that *Radix Pseudostellariae* raised the CD4^+^/CD8^+^ ratio by increasing the percentage of CD4^+^ T lymphocytes while decreasing the percentage of CD8^+^ T lymphocytes [27]. Hence, it is conceivable that *Radix Pseudostellariae* in STHJ may contribute to the effect on the levels of CD4^+^ and CD8^+^ T cells and regulate their imbalance in CTX-induced immunosuppressed mice.

To further clarify the influence of STHJ on T lymphocytes, we investigated the effects of STHJ on multiple T lymphoid subpopulations, for example, Treg, CD8^+^CD122^+^ T, *γδ*T, and NKT cells. Regulatory T cells are a subtype of CD4^+^ T cells that play key roles in the development of autoimmune and other diseases [28]. Previous studies found that the function of Treg cells is regulated by several inflammatory factors, such as IL-2, IL-6, and IL-10. Among these, IL-2, a pleiotropic cytokine produced after antigen activation, is involved in the generation of immune responses. IL-2 activates Treg cells and is used to treat autoimmune diseases caused by Treg cell defects [29]. IL-6 is a cytokine secreted by many types of cells, such as T cells and B cells, and is essential for maintaining the immune system. Studies have found that IL-6 can interfere with the function of Treg cells and prevent Th17 cells from transforming into Treg cells [30]. IL-10 is a pleiotropic cytokine mainly secreted by macrophages and plays crucial functions in the initiation of autoimmune diseases. It has been shown that IL-10 activated Treg cells, leading to the inhibition of Th17 cell function [31]. In our experiments, we found that the expression of IL-2 and IL-6 in the CTX-treated mice was significantly lower than those in the control group, while the expression of IL-10 and Treg was significantly increased. Similarly, serum levels of IL-2, IL-6, and IL-10 in the CTX-treated mice were significantly different from those of the STHJ-treated mice, but there was no compelling difference in Tregs. Therefore, the induction of Treg cells by IL-2 may counteract the effects of IL-6 and IL-10 on Treg cells. Our results are in agreement with previous results from Wang et al. [32]. Using immunosuppression models, they found that the proportion of Treg cells in the model group was significantly higher than that in the control group. Moreover, treatment with *Radix Astragali* markedly upregulated the level of Treg cells. Besides *Radix Astragali*, other ingredients in STHJ may reduce its effects on Treg by downregulating the number of Treg cells.

Accumulating evidence suggests that CD8^+^CD122^+^ T cells are a type of Tregs that inhibit autoimmunity and allogeneic immunity. It was found that high levels of CD8^+^CD122^+^ Tregs significantly enhanced the clinical symptoms of experimental autoimmune encephalomyelitis (EAE) [33]. It has also been reported that administration of CD8^+^CD122^+^ T cells increased the proportion of autoimmune Graves' hyperthyroidism in a mouse model [34]. In addition, the adoptive transfer of CD8^+^CD122^+^ Tregs may prolong the survival of allografts [35]. Therefore, CD8^+^CD122^+^ Tregs are thought to be widely involved in immune regulation. Importantly, it was reported that native CD8^+^CD122^+^ Tregs are more effective than CD4^+^CD25^+^ Tregs to induce immunosuppression [36]. In the present studies, the proportion of CD8^+^CD122^+^ T cells in the CTX-treated mice was remarkably higher than that in the control mice, suggesting that CD8^+^CD122^+^ T cells play a role in immunosuppressive states. Although STHJ had no significant effect on Treg cells in the immunosuppression model, it significantly inhibited the expression of CD8^+^CD122^+^ T cells, especially at high doses. These results suggest that STHJ has a more pronounced effect on CD8^+^CD122^+^ T cells than on Treg cells.

*γδ*T cells are a subset of T cells that have traditionally been considered to mediate cytotoxic functions. However, recent studies suggested that they also play a negative role in the regulation of immune function. Several studies have demonstrated a certain degree of transcriptional overlap between conventional Tregs and inhibitory *γδ*T cells. However, the regulatory mechanisms of the inhibitory function of these two cell types have not been fully defined [37]. In our experiments, we found that the proportion of *γδ*T cells in the CTX-treated mice was very high, suggesting that *γδ*T cells play an important role in suppressing the immune function. Although STHJ had no significant effect on Treg cells in the CTX-treated mice, we found that it significantly reduced the expression level of *γδ*T cells in this group. Therefore, the activity of *γδ*T cells appears to play important roles in mediating the immune-enhancing functions of STHJ in the immunosuppressive state.

NKT cells are a group of T cell lineages with immunomodulatory activity, mainly composed of type I and type II NKT cells [38]. Both subtypes have a regulatory role in autoimmune diseases. Type I NKT cells are implicated in systemic lupus erythematosus (SLE) and influence the pathogenicity of hepatitis. In contrast, type II NKT cells confer protection in both diseases [39]. Therefore, NKT cells may have both immune enhancement and immunosuppressive effects. When stimulated by an antigen, NKT cells can produce a range of cytokines, including IL-2, IL-10, and TNF-*α* [40, 41]. These cytokines regulate immunity by activating T cells, B cells, and other immune cells. For instance, IL-2 secreted by NKT can induce CD4^+^ T cells to differentiate into Th2 cells and inhibit Treg cells [42, 43]. Activated NKT cells can trigger the production of IgM and IgG antibodies from B cells by secreting IL-21 [44, 45]. Thus, the immunomodulatory effects of NKT cells are complex. In our experiments, the proportion of NKT cells in the CTX-treated mice was significantly higher than that in the control mice, whereas STHJ significantly inhibited the expression of NKT cells, especially at high dose. Therefore, the inhibitory effect of STHJ on NKT cells plays an important role in enhancing the immune function. Moreover, NKT cells may eliminate the effect of STHJ on Treg cells in an immunosuppressive state by inhibiting the activity of Treg cells.

Apart from T lymphocytes, B lymphocytes also play an important role in autoimmune diseases. They participate in humoral immune regulation by secreting a large number of specific antibodies. Additionally, B lymphocytes regulate other cells by producing many inflammatory cytokines, such as IL-2, IL-6, IL-10, and TNF-*α*. It has been shown that B cells aggravated EAE conditions by producing IL-6 [46]. Meanwhile, it was reported that IL-10-producing B cells inhibit autoimmune diseases by activating Tregs, thereby downregulating the production of proinflammatory cytokines [47–51]. Thus, defects in B lymphocyte functions can also cause a variety of autoimmune diseases. In this study, the proportion of B cells in the CTX-treated mice was significantly higher than that in the control mice. Interestingly, low-dose STHJ had no effect on the number of B cells, but high-dose STHJ significantly increased the proportion of B cells. This means that B cells may be less sensitive to STHJ, even though they play an important role in the mechanism of STHJ in enhancing immune functions.

## 5. Conclusion

In summary, we report that the effect of STHJ on the immune system is complex because it induces changes in the expression of various lymphocytes and inflammatory factors and also modulates interactions among these factors. Based on the results of this study, we conclude that STHJ plays a positive role in immune functions, especially at high dose. We have shown that STHJ could reverse CTX-induced immunosuppression and, therefore, our experimental results provide evidence of the immunopotentiating effects of STHJ. In a future study, we would like to investigate the effects of STHJ on a number of diseases caused by low immunity or immune disorder, such as Sjögren's syndrome.

## Data Availability

The data used to support the findings of this study are available from the corresponding author upon request.

## Abbreviations

CTX: Cyclophosphamide
TCM: Traditional Chinese medicine
STHJ: Fufang Shatai Heji
NK: Natural killer cells
RA: Rheumatoid arthritis
EAE: Experimental autoimmune encephalomyelitis
SLE: Systemic lupus erythematosus
Tregs: T regulatory cells.
